# Competitive Cooperation of Hemagglutinin and Neuraminidase during Influenza A Virus Entry

**DOI:** 10.3390/v11050458

**Published:** 2019-05-20

**Authors:** Ruikun Du, Qinghua Cui, Lijun Rong

**Affiliations:** 1 College of Pharmacy, Shandong University of Traditional Chinese Medicine, Jinan 250355, China; cuiqinghua1122@163.com; 2 Shandong Provincial Collaborative Innovation Center for Antiviral Traditional Chinese Medicine, Jinan 250355, China; 3 Qingdao Academy of Chinese Medicinal Sciences, Shandong University of Traditional Chinese Medicine, Qingdao 266122, China; 4 Department of Microbiology and Immunology, College of Medicine, University of Illinois at Chicago, Chicago, IL 60612, USA

**Keywords:** influenza A virus, hemagglutinin, neuraminidase, virus entry

## Abstract

The hemagglutinin (HA) and neuraminidase (NA) of influenza A virus possess antagonistic activities on interaction with sialic acid (SA), which is the receptor for virus attachment. HA binds SA through its receptor-binding sites, while NA is a receptor-destroying enzyme by removing SAs. The function of HA during virus entry has been extensively investigated, however, examination of NA has long been focused to its role in the exit of progeny virus from infected cells, and the role of NA in the entry process is still under-appreciated. This review summarizes the current understanding of the roles of HA and NA in relation to each other during virus entry.

## 1. Introduction

Influenza viruses belong to the family Orthomyxoviridae of enveloped viruses and are classified into four types, A, B, C and the recently identified type D [1,2]. Three main types of influenza viruses (A, B and C) infect humans, with influenza A and B viruses usually cause annual epidemics, resulting in about 3 to 5 million cases of severe illness, and about 290,000 to 650,000 respiratory deaths annually worldwide [1]. Influenza A viruses from zoonotic sources can also result in occasional but devastating pandemics [3].

The influenza A virus (IAV) and type B virus (IBV) genomes each contain eight segmented, negative-sense, single-stranded viral RNA (vRNA) segments, coding for at least 11 proteins, of which hemagglutinin (HA) and neuraminidase (NA) are the major surface glycoproteins [4]. In contrast, influenza C virus (ICV) and type D virus (IDV) carry seven segments respectively, encoding only one glycoprotein, the haemagglutinin-esterase-fusion (HEF) protein, which combines both the function of HA and NA [5,6,7]. IAVs can be further classified into subtypes based on HA and NA antigenicity. To date, many combinations of 16 HA (H1-16) and 9 NA (N1-9) subtypes have been identified in wild birds, while two additional subtypes (H17N10 and H18N11) have recently been isolated in bats [8,9,10]. Two subtypes of IAVs (H1N1 and H3N2) and two antigenically distinct lineages of IBVs (Victoria and Yamagata) currently co-circulate in humans [11].

Both HA and NA interact with sialic acid (SA), which usually links to glycoproteins and glycolipids at the cell surface [12]. HA binds to SA moieties presented by cellular receptors, triggering virus entry by clathrin-mediated endocytosis, although other endocytic routes, including micropinocytosis and raft-dependent endocytosis, may also be used [13,14,15,16,17]. In contrast, NA removes SAs from cellular receptors and from newly synthesized HA and NA on nascent virions, enabling efficient release of progeny virus at the final stage of infection [18].

The antagonistic activities on SA binding suggest a close relationship between the functions of HA and NA. The role of the viral HA in attachment and infection has been well explored, however, examination of NA has largely focused on its role in the exit of progeny virus from infected cells [19]. Interestingly, increasing pieces of evidence strongly support an essential role of NA during virus entry process [19,20]. In this review, we summarize the current state of our understanding of how the interaction of HA and NA affects IAV entry.

## 2. Hemagglutinin

**Figure 1:**
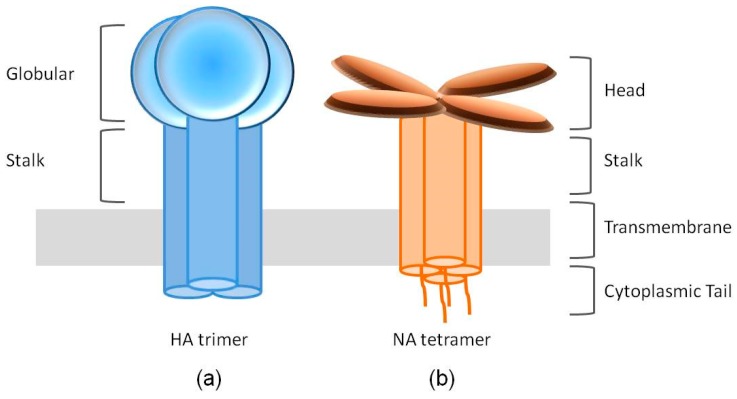
Cartoon structures of the spikes of hemagglutinin (HA) and neuraminidase (NA). (**a**) The HA spike is formed by an HA trimer. Each HA monomer contains two functional domains, the receptor binding globular domain and the helix-rich stalk domain. (**b**) NA exists as a tetramer of four identical monomers. Each NA monomer consists of four distinct structure domains, including the catalytic head, the stalk, the transmembrane region and the cytoplasmic tail.

HA is translated as an uncleaved HA0 precursor protein, assembled as a homotrimer in the endoplasmic reticulum and transported to the plasma membrane via the secretory pathway. HA0 is further cleaved into an HA1-HA2 complex by a host protease. The mature HA1-HA2 complex consists of two domains: the membrane-proximal helix-rich stalk domain, primarily composed of HA2 with some HA1 residues, and the membrane-distal globular domain (also known as the “head”) comprised of HA1, containing a SA binding pocket [21,22] (Figure 1a).

Infection of IAV is initiated when HA binds SAs on the host cell surface and triggers internalization by endocytosis. The low pH condition of the maturing endosome triggers a series of pH-induced conformational changes in HA that ultimately result in the fusion of the viral and host endosomal membranes [23]. The linkage of terminal SA to the penultimate galactose occurs via carbon 3 or carbon 6, generating two isomers α-2,3 and α-2,6 SA, respectively [24]. The SA of α-2,3 isomer predominates in the avian gut, where influenza virus replicates in avian hosts, while the α-2,6 conformer is abundant in the human upper respiratory tract, the primary site of virus replication in the human host [25,26]. Correspondingly, avian and human influenza viruses are characterized by binding α-2,3 and α-2,6 isomers respectively [27,28,29,30,31,32,33,34,35,36,37]. This receptor specificity, which is partly directed by the structural features of the HA receptor binding pocket, is one of the most important determinants that influence host tropism, including interspecies adaptation and transmissibility [38,39].

The receptor binding profiles of 18 HA subtypes are different, moreover, subtle changes in the architecture of the SA-binding pocket of HA may result in altered SA-linkage preference [33,34]. For example, human H2 and H3 strains containing leucine at position 226 favor α-2,6-linked SA, whereas avian strains containing glutamine at this position display preferential binding to α-2,3 isomer [40,41]. In addition, amino acids at positions 189, 193, 194, 216, 198 211 and 222 may also influence the architecture of the pocket region, and have been shown to be important for SA binding and specificity [37,42,43,44].

## 3. Neuraminidase

NA is assembled as homotetramers when embedded in the envelope of the virus, with each of the monomers folding into four distinct structure domains: the cytoplasmic tail, the transmembrane region, the stalk and the catalytic head [19] (Figure 1b). The cytoplasmic tail, of which the sequence is nearly 100% conserved across all IAV subtypes, plays a critical role during NA transport and incorporation [45,46,47]. The N-terminal hydrophobic transmembrane domain, however, contains a variable sequence of amino acids spanning residues 7–29 and provides signals for translocation to the apical membrane and lipid raft association [48]. The stalk domains of different NA subtypes vary considerably in the number and sequence of amino acid residues, therefore, in the length, depending on which the NA tetramers may protrude slightly more or less above the viral envelope than the HA trimers. These differences can impact the NA enzymatic activity and virulence [45,49,50]. The catalytic head domain possesses the ability to cleave SA from nearby membrane glycoproteins to prevent virus trapping [51,52].

The function of NA the during release of the newly budded virions from host cells has long been well demonstrated, however, the roles of NA in IAV entry remain under-appreciated [20,53]. NA is involved in IAV entry likely in two ways: first, NA may play a role in direct receptor binding, complementing the receptor-binding function of HA; second, NA may help to release virions bound to the sialylated “decoy” receptors, facilitating virus movement on cell surface, therefore enabling HA access to SA expressed by entry receptors and subsequent efficient endocytosis [19,20].

### 3.1. Receptor Binding

In addition to the NA catalytic site, which binds SA and its analogues, various NA proteins, including N1, N2, N5, N6 and N9, have been reported to possess a second receptor binding site (SRBS), which is also referred to as the hemadsorption site [54,55,56,57,58]. Although these NA containing SRBSs are predominantly from avian sources, influenza A (H7N9) virus containing SRBS in the N9 NA emerged as a human infection in March 2013 [59]. Moreover, it has been demonstrated that a T401A substitution within the SRBS of N9 NA may be essential for the acquisition of altered HA receptor-binding properties and contributed to the spread of the novel H7N9 viruses [58].

The NAs of human H3N2 strains acquired an SA binding property [60,61,62]. However, in contrast to the SRBS, the receptor binding function of these N2 NAs resides in the same site with the catalytic site; mutations at threonine 148, histidine 150 or aspartate 151 near the catalytic site of these NAs contributes to the acquisition of receptor binding, and the NA mediated agglutination could be inhibited by NA inhibitors [60,61,62]. Yet the catalytic and receptor binding sites seem not to be identical since the relative sensitivity of the two functions to NA inhibitors varies [60]. The biological role of the NA catalytic site-associated receptor binding function for these H3N2 isolates remains unclear, but the fact that entry of MDCK cells with viruses having D151G substitution can be blocked by NA inhibitors suggests that it is an important function [63].

### 3.2. Catalytic Activity during Virus Entry

It has been well accepted that NA activity and cleavage of SA enables movement of the virion through airway mucus during infection [64]. The sialylated mucins within airway mucus may serve as a trap by presenting decoy receptors to which influenza virus binds, followed by mucociliary clearance [65]. Previous studies demonstrated that IAV interacts with the secreted mucus on human airway tissue, and bead-bound mucins inhibit the NA cleavage of the substrate [66]. Moreover, NA inhibitors have been shown to block IAV entry into cell cultures of human airway epithelium, while exogenous NA enhanced virus passage through the mucus layer [67,68]. This suggests that the catalytic activity of NA is essential to remove decoy SAs presented on mucins so that the virus can get access to functional receptors on the target cell surface.

When it comes to infection of target cells, not every binding attempt results in success infection. Most SA associated molecules bind to HA but could not mediate endocytosis of IAV. The infection of IAV, however, is initiated by HA binding to specific IAV entry receptor proteins, such as the killer-activating proteins NKp44 and NKp46 in natural killer cells, the epidermal growth factor receptor in lung epithelial cells, and the recently identified voltage-dependent Ca^2+^ channel Ca_v_1.2 [69,70,71,72,73,74]. Therefore, most attached virions appear to require migration on the cell surface until meeting an entry receptor that subsequently mediates virus internalization.

**Figure 2:**
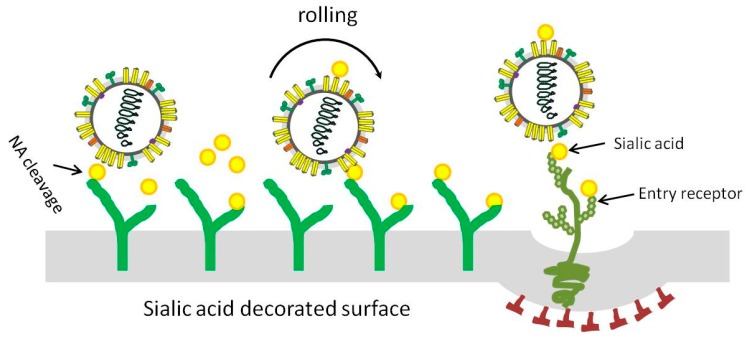
Representative model for influenza A virus moving on sialic acid (SA) decorated surface. In order to infect a target host cell, the virus might need to move either across airway mucus to reach the target cell or on the cell surface to access an entry receptor. After viral attachment, the NA could degrade SAs near the attachment site, resulting in reduced SA density; the SA density gradient promotes virus moving until successful infection occur.

The catalytic activity of NA is crucial during virus migration, and HA and NA competitively cooperate as motile machinery to move IAV particles on the cell surface [75]. As shown in Figure 2, after binding of IAV to the SA decorated surface via HA, the NA may degrade SAs near the attachment site, creating a receptor density gradient that enables the rolling of virus particles on the surface. The recent development of a biolayer interferometry assay was used to study the dynamic and motile interaction of HA/NA-receptor during the initial infection of IAV and demonstrated the contribution of NA to virus-receptor binding and NA-dependent rolling on receptor-containing surfaces [76].

### 3.3. Other Entry Steps

Using a cell-cell fusion assay, NA has also been shown to enhance the HA-mediated membrane fusion process, facilitating virus entry [77]. However, it seems that the enhancement of fusion and infectivity by NA is related to desialylation of the virion expressed HA [77].

When the NA genes from influenza H9N2, H5N1 or A(H1N1) pdm09 virus was respectively expressed on a PR8 background, the replication kinetics, both in vitro and in vivo, were not affected, but different effects on infection initiation, virus release and fusion of infected cells were observed, implicating a role for NA during the early stage of infection [78]. Quantitative proteomics analysis further demonstrated that many proteins postulated to be involved in cell-cell fusion were up-regulated in the cells infected with rPR8-H5N1NA or rPR8-H9N2NA viruses than the cells infected with wild-type virus, although the full mechanism remains to be explored [79].

## 4. Functional Balance between HA and NA

With respect to the complementary and opposite effects of HA and NA on SA binding, it is imperative that the relative activity of the two proteins is balanced, preventing the activity of one from overwhelming the role of the other; i.e., HA and NA competitive cooperation during the infection initiation and release of the newly formed virions. Three situations usually affect HA/NA balance: (i) adaptation to a new SA expression pattern after interspecies transmission occurs; (ii) antigenic drift, and (iii) presence of antivirals targeting either HA or NA [80]. Of note that the receptor-binding and receptor-destroying activities of HEF proteins of ICV and IDV should match accordingly. Since the recent study emphasized that ICV may infect the lower respiratory tract, and cause more severe disease and periodic outbreaks, while IDV also poses a potential threat to human health as an emerging pathogen, it is important to understand the characteristics of HEF proteins regarding the functional balance [81,82].

There is no straightforward method to determine the HA/NA balance of IAVs, and the most frequent method is to study each glycoprotein (HA or NA) separately. For example, the HA affinity against different substrates (α-2,3 or α-2,6 SA) can be estimated by glycan binding assay, and the NA activity can be assessed by different methods including chemiluminescence, fluorescence and glycan array assay [83,84,85]. However, not only the relative HA binding affinity and NA enzymatic activity to SA, but also several other critical factors comprehensively contribute to HA/NA balance, and the virus fitness is tuned in a much more complicated and finer way.

### 4.1. Relative HA Binding Affinity and NA Activity

All subtypes of H1-H16 and N1-N9 can be found amongst wild birds, however, only limited combinations of HA/NA occur frequently in nature whereas others rarely emerge, not to mention those infecting humans [86,87,88,89]. Laboratory attempts to produce viable reassortant viruses bearing particular subtype combinations also failed [90]. These results suggest some stringent requirements for the match between HA and NA.

**Figure 3:**
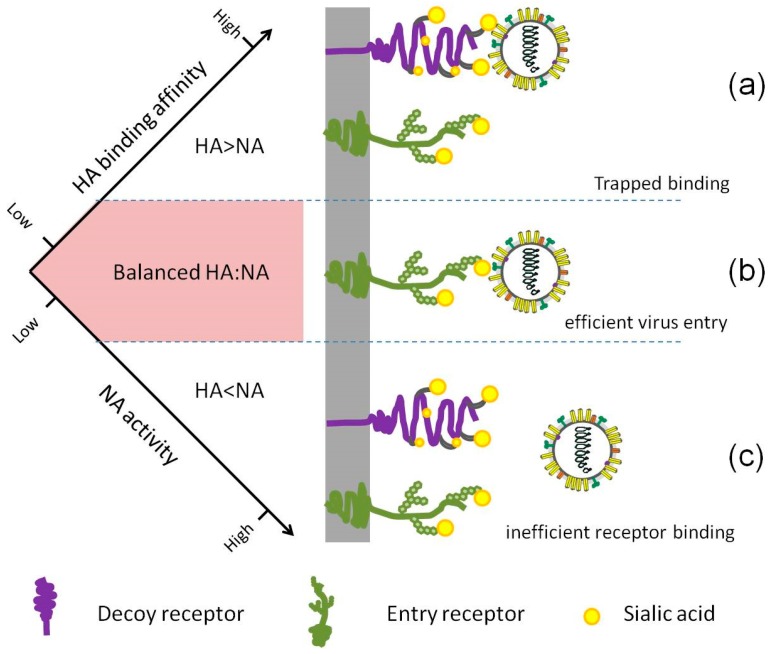
The relative HA binding affinity and NA activity need to be balanced for efficient entry. (**a**) If the HA and NA are mismatched and the NA activity is suboptimal, the virus may remain bound to decoy receptors, blocking virus movement and entry. (**b**) Efficient cleavage of SA from decoy receptors by NA enables HA access to the right entry receptors, followed by efficient endocytosis. (**c**) In the case that NA activity is too strong when compared to HA binding affinity, every binding attempt of the virus via HA will be disrupted by NA cleavage, resulting in failed binding.

Neither HA binding affinity nor NA activity alone can guarantee a successful infection, replication or transmission, but a real stable balance between the HA and NA contributes good viral fitness (Figure 3). Interestingly, several extreme cases have been reported. Upon treatment with NA inhibitor drugs, several H3N2 virus isolates from the patients were shown to have little or no NA activity, in correspondence with which a weak binding HA was found [91,92]. Hooper and Bloom created a virus in the laboratory by introducing a G147R mutation in NA, paired with binding-deficient HA, the receptor-binding activity was entirely shifted from HA to NA [93].

### 4.2. HA:NA Ratio

The relative HA binding affinity and NA activity are the foremost determinants for virus infection into a cell. In addition, a number of physical characteristics of the virus that can influence the HA/NA balance are also important. For example, the ratio of HA-to-NA is one critical factor. On average, ~300–400 HA spikes and ~40–50 NA spikes are present on the surface of a virion, and the excess of HA over NA may compensate to the weak affinity of HA for SAs by forming more connections to form a stable interaction, and vice versa [49,94].

The noncoding regions (NCRs) of the eight segmented viral RNAs (vRNAs) of IAV consist of the highly conserved promoter region and the nonconserved segment-specific NCRs at both the 3′ and 5′ ends [95,96]. Nucleotides in the nonconserved segment-specific region are highly conserved both in the sequence and the length for the same segment of different IAV strains, except for the two subtype-determinant HA and NA segments, which are further subtype-specific [97]. Although the diversity in the subtype-specific NCRs of HA and NA segments has received little attention, they are proposed to affect vRNA synthesis, protein expression and genome packaging, which might subsequently influence the amounts of HA and NA on the virion surface, as well as HA:NA ratio [98,99,100,101]. This hypothesis is supported by the recent findings which show that HA NCR plays an important role in vRNA transcription and virus infectivity [102,103].

### 4.3. NA Accessibility

The ability of NA to access the cell surface SAs is also essential for the functional HA/NA balance. Variable lengths of the NA stalk region can have a significant impact on the accessibility, although the mechanism is yet to be fully understood. Using reverse genetic techniques, a series of NA mutations differing only in the stalk length were generated, and studies showed that altered stalk length did not interfere with NA activity to cleave fetuin or a small substrate in vitro, but the increased stalk length enhanced virus replication accordingly [104,105]. It is commonly accepted that reduced stalk length results in a diminished height of NA, and the towering HA may thus block the shorter NA from gaining access to the receptor SAs [106]. Oppositely, however, reduced NA accessibility may enhance virulence and transmission in some situations. It has been reported that NA stalk truncation has arisen from an evolutionary adaptation of avian IAVs from wild aquatic birds to domestic poultry [50,107,108,109,110]. The 2009 pandemic virus A(H1N1)pdm09 with truncated NA stalk showed greater lethality in mice and virulence in ferrets compared to the untruncated counterpart [111]. It is possible that in these situations, the reduced NA accessibility might reflect the reduced HA binding affinity or enhanced NA activity.

The distribution of NA on the virion may also affect the NA accessibility. Cryoelectron tomography studies have shown that IAV virions can be sorted into five classes based on their appearance in tomograms, and the NA tetramer exists mainly in two manners: the single NA spikes surrounded by HA, and local clusters of NA [49]. Altered virion morphology and NA distribution may potentially influence viral entry, as well as viral fitness [112].

## 5. Conclusions

The complementary and antagonistic effects of HA and NA on SA interaction suggest that HA and NA competitively cooperate with each other, instead of a simplistic view that HA is responsible for virus entry while NA is involved in viral particle release, in the viral life cycle. However, the effect of the functional HA/NA balance is still poorly understood. Current antiviral therapies mainly target NA, while recent approaches to new influenza therapy include monoclonal antibodies targeting the HA [113,114]. Caution should be exercised in these approaches since it is possible that HA binding inhibitors may re-balance HA to reduce NA activity mediated by NA inhibitors, and compensating mutations in the NA may allow escape from HA inhibition and vice versa. Thus further studies to investigate the interactive roles of HA and NA in the viral entry of influenza viruses have implications in the basic biology of influenza replication and pathogenesis and in the development of therapeutics and vaccines.
